# Long noncoding RNA MALAT1 may be a prognostic biomarker in *IDH1/2* wild-type primary glioblastomas

**DOI:** 10.17305/bjbms.2019.4297

**Published:** 2020-02

**Authors:** Omer Gokay Argadal, Melis Mutlu, Secil Ak Aksoy, Hasan Kocaeli, Berrin Tunca, Muhammet Nafi Civan, Unal Egeli, Gulsah Cecener, Ahmet Bekar, Mevlut Ozgur Taskapilioglu, Cagla Tekin, Gulcin Tezcan, Sahsine Tolunay

**Affiliations:** 1 Department of Neurosurgery, Faculty of Medicine, Uludag University, Bursa, Turkey; 2 Department of Medical Biology, Faculty of Medicine, Uludag University, Bursa, Turkey; 3 Faculty of Medicine, Uludag University, Bursa, Turkey; 4 Institute of Fundamental Medicine and Biology, Kazan Federal University, Kazan, Russia; 5 Department of Pathology, Faculty of Medicine, Uludag University, Bursa, Turkey

**Keywords:** Long noncoding RNA, MALAT1, biomarker, prognosis, *IDH1/2*, primary glioblastoma, isocitrate dehydrogenase

## Abstract

Primary glioblastoma (GB) is the most aggressive type of brain tumors. While mutations in isocitrate dehydrogenase (IDH) genes are frequent in secondary GBs and correlate with a better prognosis, most primary GBs are IDH wild-type. Recent studies have shown that the long noncoding RNA metastasis associated lung adenocarcinoma transcript-1 (MALAT1) is associated with aggressive tumor phenotypes in different cancers. Our aim was to clarify the prognostic significance of MALAT1 in *IDH1/2* wild-type primary GB tumors. We analyzed *IDH1/2* mutation status in 75 patients with primary GB by DNA sequencing. The expression of MALAT1 was detected in the 75 primary GB tissues and 5 normal brain tissues using reverse transcription quantitative PCR (RT-qPCR). The associations between MALAT1 expression, *IDH1/2* mutation status, and clinicopathological variables of patients were determined. *IDH1* (R132H) mutation was observed in 5/75 primary GBs. *IDH2* (R172H) mutation was not detected in any of our cases. MALAT1 expression was significantly upregulated in primary GB vs. normal brain tissues (*p* = 0.025). Increased MALAT1 expression in IDH1/2 wild-type primary GBs correlated with patient age and tumor localization (*p* = 0.032 and *p* = 0.025, respectively). A multivariate analysis showed that high MALAT1 expression was an unfavorable prognostic factor for overall survival (*p =* 0.034) in *IDH1/2* wild-type primary GBs. High MALAT1 expression may have a prognostic role in primary GBs independent of IDH mutations.

## INTRODUCTION

Glioblastoma (GB) is the most common and most aggressive primary brain tumor (World Health Organization [WHO] astrocytoma grade IV) [1]. Conventional therapies, including surgery, adjuvant radiotherapy, and pharmacotherapy (typically chemotherapy with temozolomide [TMZ]) have not improved the prognosis of GB patients, and the 5-year survival rate has remained at 4–6% [2]. GBs are classified as primary or secondary according to their formation [3,4]. The two GB types are characterized by different genetic alterations, and mutations in isocitrate dehydrogenase (IDH) genes are most commonly used to discriminate between primary and secondary GBs [5-7]. Many studies showed that *IDH* mutations are more frequent in secondary GB (>80%) than in primary GB (<5%) and that they correlate with a better prognosis [8,9]. While a mutation in the *IDH1* gene is considered a prognostic marker in secondary GB, the effect of *IDH1/2* mutations on the prognosis of primary GB remains unclear.

In addition to *IDH* mutations, epigenetic regulation is now thought to play an important role in the development and prognosis of GB [10]. These epigenetic mechanisms include regulation by long noncoding RNAs (lncRNAs), which are noncoding transcripts of more than 200 nucleotides in length [11]. LncRNAs regulate many cellular processes, such as gene transcription and translation. Dysregulation of lncRNA expression affects cell proliferation, cell migration, tumor growth, and metastasis in several types of cancer, including brain tumors. LncRNAs have been associated with oncogenic mechanisms in oligodendroglioma, medulloblastoma, and GB. Oncogenic lncRNAs may affect glial cell differentiation and maintenance of stemness, leading to metastasis [11]. Based on differential expression patterns of lncRNAs in brain tumors they may be used as diagnostic and prognostic biomarkers as well as pharmaceutical targets [12].

Metastasis associated lung adenocarcinoma transcript-1 (MALAT1) located on chromosome 11q13 was found to be highly expressed and associated with metastasis in non-small cell lung cancer [13]. MALAT1 is involved in the regulation of genes related to proliferation, apoptosis, and metastasis of tumor cells [14]. As an oncogene, MALAT1 can induce migration and invasion of tumor cells. MALAT1 overexpression in glioma tissues was positively correlated with grade and tumor size [14]. Recent studies reported that MALAT1 is one of the most significantly upregulated lncRNAs in various tumors, including gliomas [15-17]. However, the significance of MALAT1 in GB prognosis, especially in a homogenous group of patients with primary GB, is unclear.

Based on the available evidence for aberrant expression of MALAT1 in cancer, we hypothesized that dysregulation of MALAT1 plays an important role in the pathogenesis of primary GB. Therefore, in the current study, we analyzed MALAT1 expression in patients with *IDH1/2* wild-type primary GB.

## MATERIALS AND METHODS

### Patients

This retrospective study included 75 patients with a diagnosis of primary GB, who were treated at the Department of Neurosurgery of the Uludag University Hospital between 2005 and 2016. Tumor tissue samples were obtained by surgical resection before treatment (radiation or chemotherapy). Surgical procedures were performed by neurosurgeons with the use of microscope (Zeiss OPMI Pentero Carl Zeiss Inc., Oberkochen, Germany). The aim of the surgery was gross total resection whenever possible, since the maximal extent of resection is correlated with longer recurrence-free period and overall survival (OS) of patients. Intraoperative fluorescence techniques that are known to help maximize the extent of resection, such as those using 5-aminolevulinic acid (5-ALA), indocyanine green, or fluorescein sodium, were not routinely used in any of the cases. Patients who underwent only stereotactic biopsy, who had a family history of glioma, who had a coexisting malignancy, or who died for different reasons within the first 30 days after surgical resection were excluded from the study. Tumor samples were examined and confirmed at the Department of Pathology of the Uludag University and classified according to the WHO criteria. This study was approved by the Uludag University Ethics Committee (ethical number 2017-13/98).

### DNA extraction

Expert pathologists identified tumor locations in formalin-fixed paraffin embedded (FFPE) tissues. Paraffin was removed with xylene and 95% ethanol, and DNA was isolated from the fixed tissues using the DNeasy FFPE Mini Kit (Qiagen, Germany), according to the manufacturer’s protocol. The amount and concentration of extracted DNA in a 4-µl volume of each sample were measured using a Beckman Coulter DU-730 spectrophotometer (Beckman Coulter Inc., CA, US). Only high-quality DNA samples were used for subsequent analyses, i.e., with the absorbance ratio between 1.9 and 2.1.

### *IDH1/2* mutation analysis

Point mutations in the *IDH1* and *IDH2* genes were analyzed by direct sequencing of polymerase chain reaction (PCR) products. The genomic regions of R132H mutation in *IDH1* and R172H in *IDH2* represent 254-bp and 345-bp long fragments, respectively and span the catalytic domains of corresponding proteins. We used the following primers for direct sequencing of the exon 4 in both genes: *IDH1* forward 5′-TGAGAAGAGGGTTGAGGAGTT-3′ and reverse 5′-AACATGCAAAATCACATTATTGCC-3′. *IDH2* forward 5′-CACGCTGAAGAAGATGTGGAA -3′ and reverse 5′-CAGAGACAAGAGGATGGCTA-3′. PCR was performed in a 20-µl reaction volume containing 1.25 µl of each forward and reverse primer, 10 µl Master mix buffer, 4.5 µl dH_2_O, and 3 µl DNA. The PCR conditions were as follows: Denaturation at 95°C for 2 min and at 94°C for 12 sec, 40 cycles of amplification with annealing at 53°C for 30 sec and extension at 72°C for 59 sec, and final extension at 72°C for 7 min.

### RNA extraction and reverse transcription quantitative PCR (RT-qPCR) analysis of MALAT1 expression

Total RNA was extracted from FFPE tissues using the RNeasy FFPE Mini Kit (Qiagen, Germany) to determine the expression levels of MALAT1. The quantity and concentration of extracted RNA were evaluated in 4 µl of each sample using a Beckman Coulter DU-640 spectrophotometer. The absorbance ratio of extracted RNA samples was between 1.8 and 2.2. The cDNA synthesis was performed using the ProtoScript M-MuLV Fist Strand cDNA Synthesis Kit (New England Biolabs, MA, US). The PCR conditions for cDNA synthesis were as follows: 25°C for 10 min, 37°C for 2 h, and 85°C for 5 min. LncRNA expression was analyzed using an ABI StepOnePlus^TM^ real-time PCR (Applied Biosystems, CA, US). The expression levels of MALAT1 (Hs00273907_s1) were normalized to the levels of glyceraldehyde-3-phosphate dehydrogenase (GAPDH) gene (Hs.544577).

### Statistical analysis

The pathogenic significance of *IDH1* (R132H) and *IDH2* (R172H) variants was determined using ClinVar. The associations between MALAT1 expression, *IDH1/2* mutation status, and clinicopathological variables of patients were analyzed using IBM SPSS Statistics for Windows, Version 23.0 (IBM Corp., Armonk, NY). The Chi-square test and Cox regression were used for multivariate analysis. The effects of *IDH1/2* mutation status and MALAT1 expression on patient survival were determined by Kaplan-Meier analysis using MedCalc Statistical Software version 12.4.0 (MedCalc Software bvba, Ostend, Belgium).

## RESULTS

### Clinicopathological features of patients

A total of 75 patients with primary GB were analyzed in this retrospective study; 34 (45.3%) were female and 41 (54.7%) were male. The age at GB presentation ranged from 31 to 76 years (median of 56 years). Tumors were localized in the right side of the brain in 36 (48.0%) patients and in the left side in 39 (52.0%) patients. All patients were classified as grade IV based on the WHO criteria for grading of infiltrating gliomas. The rate of gross total resection was 27% as determined by contrast-enhanced magnetic resonance imaging (MRI). The clinicopathological features of patients are summarized in Table 1.

**TABLE 1:**
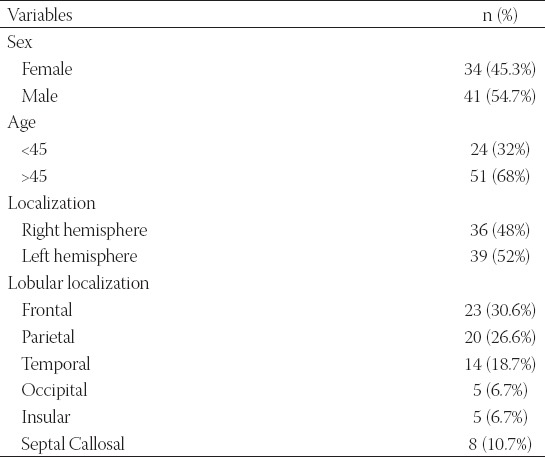
Clinicopathological features of primary glioblastoma (GB) patients (N=75)

### Prevalence of *IDH1/2* mutations in primary GB

**FIGURE 1:**
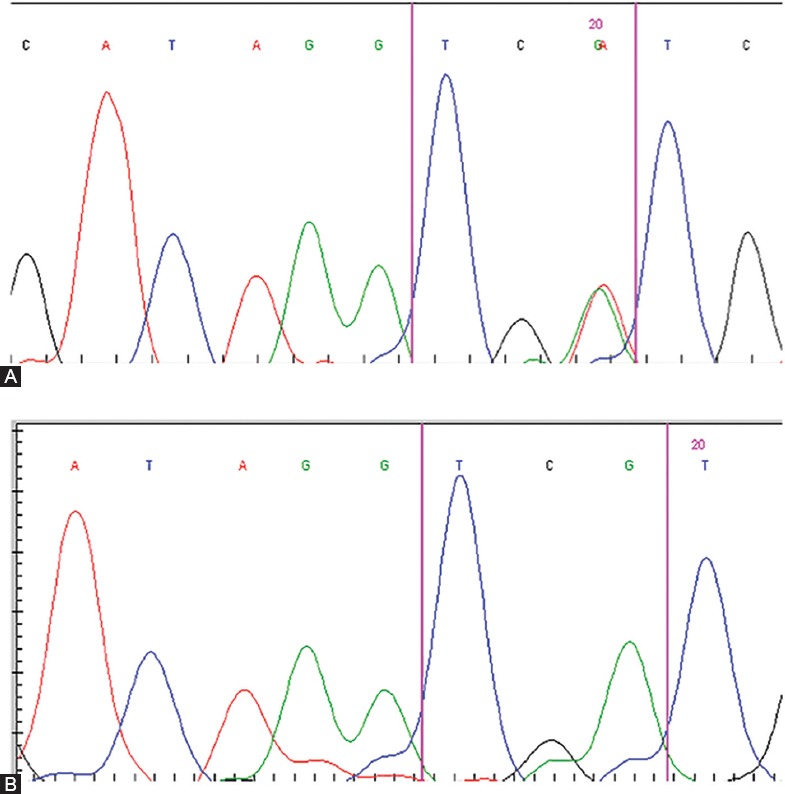
The *IDH1* R132H mutation was analyzed by direct sequencing in primary GB tumors. (A) Presence of the R132H mutation; (B) Absence of the R132H mutation. IDH: Isocitrate dehydrogenase; GB: Glioblastoma.

**FIGURE 2:**
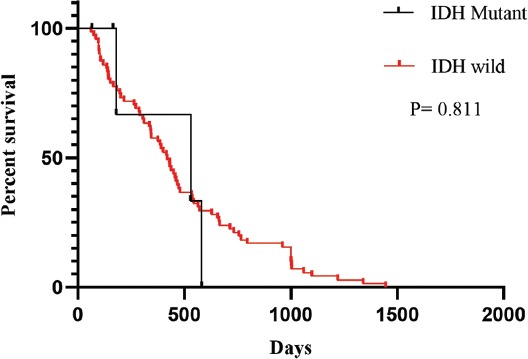
The Kaplan-Meier survival analysis showed no effect of the *IDH1* R132H mutation on the overall survival of patients with primary GB. IDH: Isocitrate dehydrogenase; GB: Glioblastoma.

Seventy-five primary GB tumor samples were assessed by DNA sequencing to identify *IDH1/2* mutations. The *IDH1* R132H mutation was observed in 5/75 (6.7%) GB patients (Figure 1). The *IDH2* R172H mutation was not detected in any of our cases. There were no significant correlations between the IDH1 mutation and patient age, gender, or tumor localization (Table 2). The Kaplan-Meier survival analysis showed no effect of the *IDH1* mutation on the OS of GB patients (Figure 2).

**TABLE 2:**
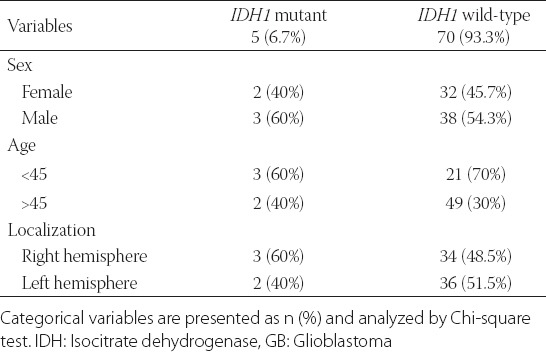
Analysis of the association of *IDH1* (R132H) mutation with sex, age, or tumor localization in primary GB patients (N=75)

### Expression of MALAT1 in primary GB

**FIGURE 3:**
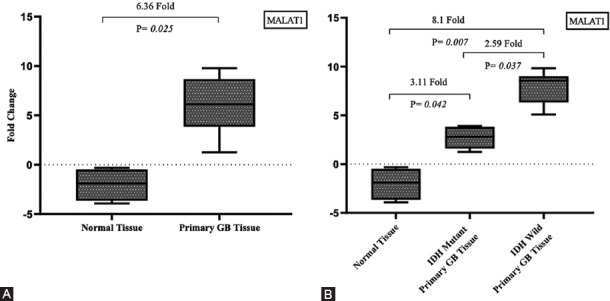
Expression of MALAT1 in primary GB. (A) The expression of MALAT1 was 6.36-fold increased in primary GB tumors compared with normal brain tissues. (B) The expression of MALAT1 was 2.59-fold increased in *IDH1/2* wild-type primary GBs vs. *IDH1* mutant. IDH: Isocitrate dehydrogenase, GB: Glioblastoma, MALAT1: Metastasis associated lung adenocarcinoma transcript-1.

To determine the diagnostic and prognostic significance of MALAT1 in primary GB we analyzed its expression in 5 unrelated *IDH1* mutant primary GBs, 70 *IDH1/2* wild-type primary GBs, and 5 normal brain tissues by RT-qPCR. The expression of MALAT1 was 6.36-fold increased in primary GB tumors compared with normal brain tissues (Figure 3A; *p* = 0.025). Furthermore, the expression of MALAT1 was 2.59-fold increased in *IDH1/2* wild-type vs. *IDH1* mutant primary GBs (Figure 3B; *p* = 0.037).

### Expression of MALAT1 in *IDH1/2* wild-type primary GB

**FIGURE 4:**
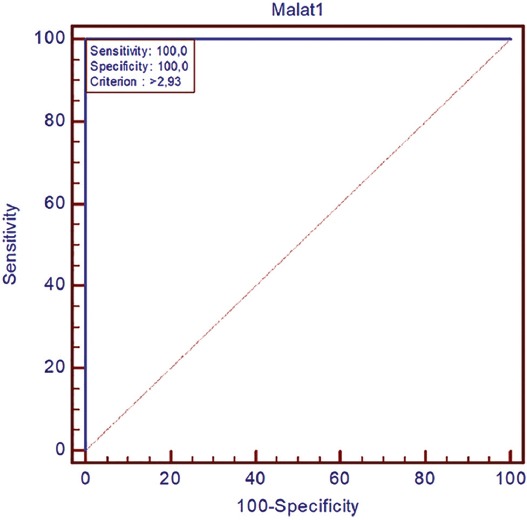
Based on the cutoff value of MALAT1 fold change, 39/70 (56%) IDH1/2 wild-type primary GBs were identified as upregulated and 31/70 (44%) as downregulated (r ≥ 2.93; *p* < 0.001). IDH: Isocitrate dehydrogenase, GB: Glioblastoma, MALAT1: Metastasis associated lung adenocarcinoma transcript-1.

**FIGURE 5:**
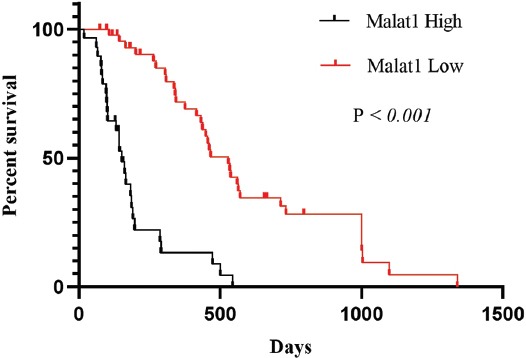
Kaplan–Meier analysis of overall survival in patients with *IDH1/2* wild-type primary GB in relation to MALAT1 expression. Patients with *IDH1/2* wild-type primary GB and upregulated MALAT1 expression had a significantly shorter survival time compared with those with downregulated MALAT1 expression. IDH: Isocitrate dehydrogenase, GB: Glioblastoma, MALAT1: Metastasis associated lung adenocarcinoma transcript-1.

The expression of MALAT1 was 8.21-fold increased in *IDH1/2* wild-type primary GB tumors compared with normal brain tissues (Figure 3B, *p* = 0.007). Based on the cutoff value of MALAT1 fold change, 39/70 (56%) *IDH1/2* wild-type primary GBs were identified as upregulated and 31/70 (44%) as downregulated (Figure 4; r ≥ 2.93; *p* < 0.001). We further analyzed clinicopathological features in relation to these two groups of GB patients. There was no significant association between MALAT1 expression and gender. However, increased MALAT1 expression was associated with lower age and tumor localization (*p =* 0.032 and *p =* 0.025, respectively). The expression of MALAT1 was higher in left GB tumors (*p* = 0.025) and it was especially increased in the insular cortex compared with other brain lobes (*p* = 0.004; Table 3). The Kaplan–Meier analysis with log-rank test showed that patients with *IDH1/2* wild-type primary GB and upregulated MALAT1 expression had a significantly shorter survival time compared with those with downregulated MALAT1 expression (*p* < 0.001; Figure 5).

**TABLE 3:**
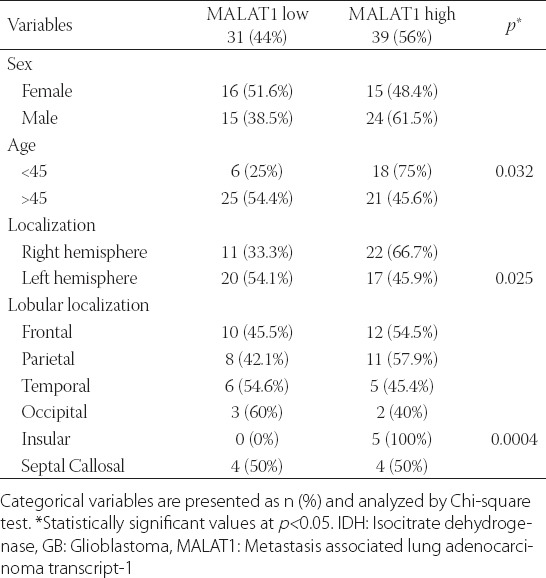
Analysis of the association of MALAT1 expression with clinicopathological features of patients with *IDH1/2* wild-type primary GB (n=70)

### Multivariate analysis

The multivariate cox regression model that included age, tumor localization, *IDH1/2* mutation status, and MALAT1 expression status showed that high MALAT1 expression was a significant predictor of poor prognosis in patients with primary GB, with the median follow-up period of 60 months (N = 75; *p* = 0.034; Table 4). Our results suggest that MALAT1 has an oncogenic role in primary GB tumors.

**TABLE 4:**
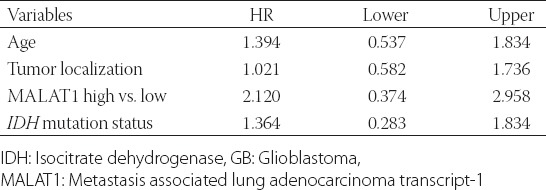
Multivariate Cox regression analysis of overall survival in patients with primary GB (N=75)

## DISCUSSION

The main aim of this study was to determine the prognostic significance of MALAT1 expression in primary GB in general and in relation to *IDH1/2* mutation status. While *IDH1/2* mutations are rare in primary GB they are frequently observed in secondary GB. According to the 2016 WHO criteria, IDH mutations can be used to discriminate between primary and secondary GBs. However, little is known about the prognostic significance of *IDH1/2* mutations in primary GB [18]. We detected the *IDH1* R132H mutation in 6.7% of patients with primary GB. Previous studies reported *IDH1* mutations in 5–12% of primary GBs. Furthermore, they showed that the presence of an IDH mutation in gliomas is associated with relatively better prognosis and tumor occurrence at a younger age [19-22]. In 118 Chinese patients with primary GB the *IDH1* R132H mutation was limited as a prognostic factor, according to the multivariate regression model that included gender, age, preoperative Karnofsky Performance Score (KPS) score, extent of resection, TMZ chemotherapy, and Ki-67 protein expression level [23]. To the best of our knowledge, only one other study investigated *IDH1* mutation status of primary GBs in a Turkish population, and they also showed that there is a lack of association between the *IDH1* R132H mutation and OS [24]. In our study there was no significant association between OS or any of clinical features and *IDH1* mutation status in patients with primary GB.

Dysregulation of MALAT1 is associated with metastasis and the risk of tumor recurrence after treatment in various cancer types [25]. There is accumulating evidence that MALAT1 is overexpressed in gliomas [17]. However, the prognostic significance of MALAT1 in primary GB is unclear. In the present study, we found that MALAT1 was upregulated in primary GB. Nevertheless, previous studies reported contradictory results on the expression profile of MALAT1 in GB. Fawzy et al. showed that MALAT1 expression was downregulated in Egyptian patients with GB and that lower MALAT1 expression was associated with tumor recurrence, lower OS, and shorter disease-free survival (DFS) [26]. Han et al. reported that MALAT1 acts as a tumor suppressor in glioma cells by downregulation of matrix metalloproteinase-2 (MMP2) and inactivation of the extracellular signal-regulated kinase (ERK)/mitogen-activated protein kinase (MAPK) pathway [27]. On the other hand, Chen et al. demonstrated high MALAT1 expression in GB patients that was associated with poor response to TMZ treatment and lower survival. In addition, they showed that MALAT1 is upregulated in TMZ resistant GB cell lines [27]. In our study, upregulated MALAT1 expression was associated with shorter OS in patients with *IDH1/2* wild-type primary GB. Furthermore, high MALAT1 expression was associated with younger age and left-sided GB localization in our group. Particularly, MALAT1 expression was significantly increased in the insular lobe compared with other lobes. In the previous studies, MALAT1 expression was not associated with tumor localization. The association of MALAT1 expression with clinicopathological features of primary GB patients remains to be determined. Additionally, the expression of lncRNAs in tumors may differ between patients of different ethnic origin. Thus, more clinical studies are needed to determine the functional role of MALAT1 in primary GB.

## CONCLUSION

The pathogenesis of primary GB is not completely understood and, currently, there are no curative therapies available for this subgroup of GB. Thus, it is important to identify novel molecular biomarkers or therapeutic targets for this relentless disease. We showed that high MALAT1 expression was an independent poor prognostic factor for patients with *IDH1/2* wild-type primary GB suggesting that MALAT1 may be a potential prognostic marker and therapeutic target in primary GB patients.
